# Optical coherence tomography angiography reveals insights into complementary vascular and neurodegenerative mechanisms in multiple sclerosis

**DOI:** 10.1093/braincomms/fcag007

**Published:** 2026-01-10

**Authors:** Charmaine Hiu-Ying Yam, Danuta M Sampson, Andreia Marques Elias, Jed Wingrove, Baris Kanber, Ronja Christensen, Pryanka Sood, Riccardo Nistri, Anna He, Alyssa A Toorop, Elena Panella, Dimitrios Champsas, Suraya Mohamud, Weaam Hamed, Ferran Prados Carrasco, Frederik Barkhof, Ahmed T Toosy, Olga Ciccarelli

**Affiliations:** Queen Square MS Centre, Department of Neuroinflammation, UCL Queen Square Institute of Neurology, Faculty of Brain Sciences, University College London, London WC1B 5EH, UK; Neurosciences Institute, Cleveland Clinic London, London SW1X 7HY, UK; Queen Square MS Centre, Department of Neuroinflammation, UCL Queen Square Institute of Neurology, Faculty of Brain Sciences, University College London, London WC1B 5EH, UK; Centre for Ophthalmology and Visual Science (Incorporating Lions Eye Institute), The University of Western Australia, Perth 6009, Australia; Queen Square MS Centre, Department of Neuroinflammation, UCL Queen Square Institute of Neurology, Faculty of Brain Sciences, University College London, London WC1B 5EH, UK; Queen Square MS Centre, Department of Neuroinflammation, UCL Queen Square Institute of Neurology, Faculty of Brain Sciences, University College London, London WC1B 5EH, UK; Queen Square MS Centre, Department of Neuroinflammation, UCL Queen Square Institute of Neurology, Faculty of Brain Sciences, University College London, London WC1B 5EH, UK; UCL Hawkes Institute, Department of Medical Physics and Biomedical Engineering, University College London, London WC1E 6BT, UK; Queen Square MS Centre, Department of Neuroinflammation, UCL Queen Square Institute of Neurology, Faculty of Brain Sciences, University College London, London WC1B 5EH, UK; Queen Square MS Centre, Department of Neuroinflammation, UCL Queen Square Institute of Neurology, Faculty of Brain Sciences, University College London, London WC1B 5EH, UK; Queen Square MS Centre, Department of Neuroinflammation, UCL Queen Square Institute of Neurology, Faculty of Brain Sciences, University College London, London WC1B 5EH, UK; Department of Neurology, Great Ormond Street Hospital for Children, London WC1N 3JH, UK; Queen Square MS Centre, Department of Neuroinflammation, UCL Queen Square Institute of Neurology, Faculty of Brain Sciences, University College London, London WC1B 5EH, UK; Department of Clinical Neuroscience, Karolinska Institute, Stockholm 171 77, Sweden; Department of Neuroscience, Monash University Melbourne, Melbourne 3181, Australia; Department of Rehabilitation Medicine, Amsterdam UMC, Amsterdam 1105 AZ, The Netherlands; Child Neurology and Psychiatry Unit, Systems Medicine Department, Tor Vergata University of Rome, Rome 00133, Italy; Department of Neurology, Great Ormond Street Hospital for Children, London WC1N 3JH, UK; Queen Square MS Centre, Department of Neuroinflammation, UCL Queen Square Institute of Neurology, Faculty of Brain Sciences, University College London, London WC1B 5EH, UK; Queen Square MS Centre, Department of Neuroinflammation, UCL Queen Square Institute of Neurology, Faculty of Brain Sciences, University College London, London WC1B 5EH, UK; Queen Square MS Centre, Department of Neuroinflammation, UCL Queen Square Institute of Neurology, Faculty of Brain Sciences, University College London, London WC1B 5EH, UK; UCL Hawkes Institute, Department of Medical Physics and Biomedical Engineering, University College London, London WC1E 6BT, UK; eHealth Center, Universitat Oberta de Catalunya, Barcelona 08018, Spain; Queen Square MS Centre, Department of Neuroinflammation, UCL Queen Square Institute of Neurology, Faculty of Brain Sciences, University College London, London WC1B 5EH, UK; Brain Repair and Rehabilitation, Institute of Neurology, Faculty of Brain Sciences, University College London, London WC1N 3BG, UK; Radiology and Nuclear Medicine, Amsterdam University Medical Center, Amsterdam 1081 BT, The Netherlands; Queen Square MS Centre, Department of Neuroinflammation, UCL Queen Square Institute of Neurology, Faculty of Brain Sciences, University College London, London WC1B 5EH, UK; Queen Square MS Centre, Department of Neuroinflammation, UCL Queen Square Institute of Neurology, Faculty of Brain Sciences, University College London, London WC1B 5EH, UK; National Institute for Health and Care Research University College London Hospitals Biomedical Research Centre, London NW1 2PG, UK

**Keywords:** optical coherence tomography angiography, retinal microvasculature, brain atrophy, multiple sclerosis, visual outcomes

## Abstract

Optical coherence tomography angiography quantifies retinal microvasculature biomarkers, offering insights into neurovascular mechanisms underlying brain damage in multiple sclerosis. This study evaluated these potential mechanisms of neurodegeneration by examining associations between optical coherence tomography and optical coherence tomography angiography metrics, brain volumes and clinical outcomes in people with multiple sclerosis. This cross-sectional study included multiple sclerosis patients from a prospective cohort. Participants underwent optical coherence tomography/optical coherence tomography angiography, vision and clinical assessments and brain MRI. Age- and sex-matched controls underwent optical coherence tomography/optical coherence tomography angiography. The OCTA Vascular Analyser toolbox was used to derive metrics that reflect superficial plexus retinal vessel density (vessel area density, vessel length density) and network complexity. Differences in optical coherence tomography angiography and optical coherence tomography (peripapillary retinal nerve fibre layer and macular ganglion cell-inner plexiform layer) metrics between controls and patient eyes and associations with brain volumes and visual outcomes were analysed using linear-mixed models, adjusted for age, sex, disease duration and optic neuritis. Vision outcome models were compared using Akaike Information Criterion. The study included 323 multiple sclerosis patients (603 eyes; 98 with prior optic neuritis) and 80 controls (147 eyes), with 267 patients undergoing brain MRI. Patients exhibited reduced vessel area density and vessel length density and thinner peripapillary retinal nerve fibre layer and macular ganglion cell-inner plexiform layer in non-optic neuritis eyes compared with controls. Optic neuritis eyes showed deviations compared with non-optic neuritis eyes. In patients, reductions in optical coherence tomography angiography and optical coherence tomography metrics were associated with smaller volumes of the primary visual cortex (calcarine cortex and occipital pole) and thalamus but reduced vessel area density/vessel length density correlated predominantly with smaller higher-order visual processing region volumes, such as the cuneus (vessel area density: β = 0.36, vessel length density: β = 0.070), inferior occipital (vessel area density: β = 0.28, vessel length density: β = 0.057) and occipital fusiform gyrus (vessel area density: β = 0.51, vessel length density: β = 0.094) (all *P* < 0.01). In contrast, peripapillary retinal nerve fibre layer thinning was associated with smaller white matter (β = 0.10, *P* = 0.008) and optic chiasm volumes (β = 293.30, *P* < 0.0001). Reduced vessel densities were more strongly associated with worse high-contrast visual acuity and colour vision than macular ganglion cell-inner plexiform layer and peripapillary retinal nerve fibre layer. Retinal microvasculature abnormalities were associated with regional grey matter atrophy in higher-order vision processing regions. We speculate that cerebral hypoperfusion—and by proxy, retinal hypoperfusion—may be mechanistically related to region-specific neurodegeneration. In contrast, peripapillary retinal nerve fibre layer thinning may reflect broader neurodegenerative processes, including Wallerian degeneration and disrupted white matter connectivity. Clinically, this manifests as impaired contrast sensitivity and colour vision. These findings underscore optical coherence tomography angiography’s potential as a complementary biomarker to optical coherence tomography in probing visual pathway integrity, highlighting its promise for assessing neurovascular pathology and progression in early multiple sclerosis.

## Teaser

Yam *et al*. compared optical coherence tomography and angiography metrics in multiple sclerosis patients and controls. They demonstrated that multiple sclerosis eyes had reduced retinal vessel densities, correlating with atrophy of higher-order visual regions and worse vision, while retinal nerve fibre layer thinning correlated with white-matter and chiasmal volume loss.

## Graphical Abstract

**Graphical Abstract:**
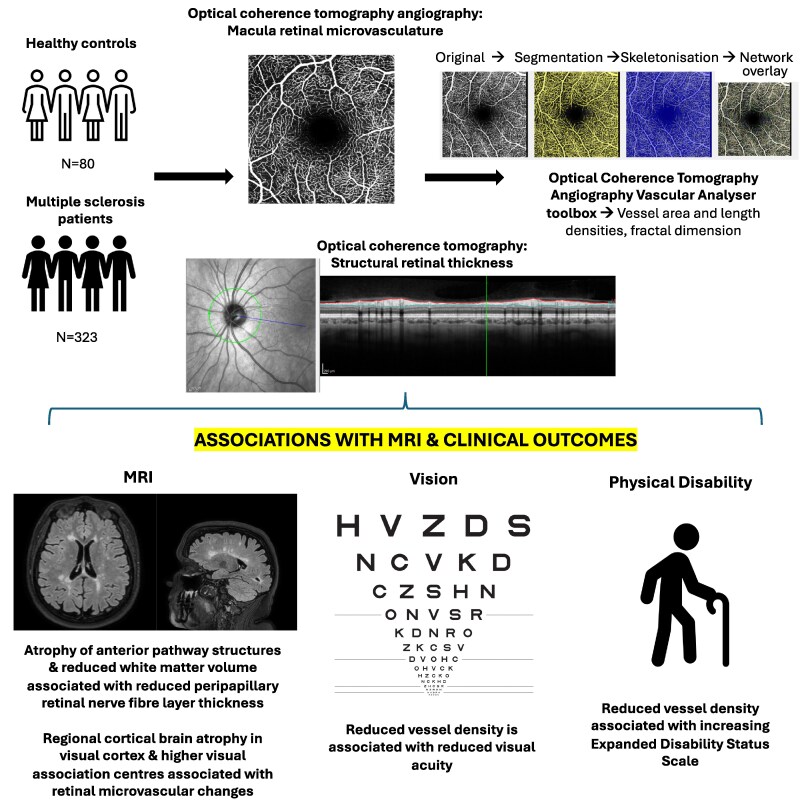

## Introduction

Optical coherence tomography angiography (OCTA) images the retinal microvasculature quickly and conveniently within a clinical setting. Unlike fluorescein angiography, OCTA does not require intravenous contrast. It detects blood flow by using motion contrast, comparing the decorrelation signals between multiple B-scans at each cross-section.^1^

Reduced retinal microvasculature density is linked to cerebral atrophy and pathological changes in neurodegenerative conditions and normal ageing.^2,3^ Genome-wide association studies have shown potential bi-directional, causal relationships between retinal vascular density, fractal dimension (FD) and cortical structure, signifying pathophysiologic interactions that could predict future neuropsychiatric disorders.^4^ Cerebral hypoxia plays a role in the pathogenesis of multiple sclerosis (MS).^5,6^ Brain imaging studies have shown reduced cortical and deep grey matter cerebral blood flow on MRI in patients with early relapsing remitting MS, even without corresponding volume loss,^7^ suggesting that cerebral hypoperfusion may precede brain atrophy. Near-infrared spectroscopy studies have demonstrated reduced microvascular haemoglobin oxygen saturation in the cerebral cortex of people with MS, particularly those with secondary progressive and high-disability relapsing remitting MS; this reduction was associated with worse motor disability, as shown by performance on the 9-hole peg test, 25-foot walk and expanded disability status scale (EDSS).^8^ Differences in the findings between groups may be attributed to the magnitude of inflammation and resultant hypoxia in relapsing patients relative to progressive patients and controls; however, authors acknowledged there may be other pathophysiological processes such as arterial-venous shunting and redirection of vascular supply that requires consideration.^8^

The role of OCTA in MS has been explored in small cohort studies. Studies have demonstrated that relapsing remitting MS patients exhibit lower superficial plexus density than clinically isolated syndrome patients and healthy controls.^9^ Additionally, OCTA improved the diagnostic accuracy of OCT in distinguishing MS non-optic neuritis eyes from healthy controls.^10^ Reduced superficial plexus densities are associated with longer disease duration and worse disability,^11^ as well as MRI markers of neurodegeneration (especially in grey matter).^12^ Despite promising evidence for OCTA as a biomarker in MS, most studies are small, with insufficient evidence to support a complementary or additive role for OCTA alongside structural OCT. This is compounded by quality control issues inherent to the technique, as well as a lack of standardization in image postprocessing, analysis and interpretation.^13^

This cross-sectional study aimed to determine whether OCTA provides insights into the mechanisms of brain volume loss and visual outcomes in people with MS. In our cohort, which included patients with relatively short disease duration and low disability largely due to high-efficacy treatment use in our centre, we explored whether OCTA could identify retinal hypoperfusion independently of overt neurodegeneration. We hypothesized that people with MS with lower physical disability and shorter disease duration exhibit evidence of retinal hypoperfusion compared with controls, quantified by OCTA, but not reflected by OCT. Furthermore, we hypothesized that OCTA would be associated with regional brain structural changes reflecting cerebral hypoperfusion and oxygen supply-demand mismatch in centres vital for visual processing (i.e. primary visual cortex and regions of the dorsal and ventral visual streams, such as occipital [e.g. occipital fusiform gyrus, lingual gyrus and the cuneus], superior/posterior parietal and temporal cortical areas),^14^ and that these associations might differ from or be stronger than those observed with OCT.

## Materials and methods

### Study design and participants

Patients who consented to be recruited into the observational, prospective, single-centre POINT-MS (Predicting Optimal INdividualized Treatment response in Multiple Sclerosis) study which enrolled patients at the National Hospital for Neurology and Neurosurgery, London, UK between 2019 and 2024, were invited to attend the hospital for an OCT/OCTA scan.^15^ Entry criteria included (i) age ≥ 18 years; (ii) diagnosis of MS according to the 2017 McDonald criteria^16^ and (iii) Initiating or changing to a new DMT within 3 months from study entry. For this study, we conducted a cross-sectional analysis of OCT^/^OCTA acquired between October 2021 and October 2023. Healthy controls (HCs) were also recruited, and inclusion criteria were adults aged 18–65 without MS or neurological diseases. Exclusion criteria were (i) known retinal pathology as per the OSCAR-IB criteria;^17^ (ii) refractive errors >6 or <−6 dioptres; (iii) optic neuritis within the last 6 months and (iv) MS or other neurological disease/condition.

Clinical information including age, sex, optic neuritis history, MS disease duration (from symptom-onset), relapses, medication history, body mass index, cardiovascular risk factors (smoking and alcohol intake) and ethnicity were collected. For HCs, age, sex, ocular and cardiovascular comorbidities and refractive error were recorded.

Participants’ written, informed consent was obtained according to the Declaration of Helsinki and local ethics committee approval was in place for the POINT-MS study (19/WA/0157, Sub_Amend_3) and Healthy control study (23655.001).

### Optical coherence tomography/optical coherence tomography angiography acquisition and analysis

POINT-MS patients were invited to undergo OCT/OCTA assessment on the day of their MRI scans and clinical assessments; if not possible, then OCT/OCTAs were obtained within 90 days of the MRI scans. Pupils were undilated for the OCT/OCTA, which was performed on a Spectralis OCT2 machine (Heidelberg, Germany) and Spectralis software V7.0.1. For the pRNFL scan, the instrument used 1024 A-scan points with a 3.45 mm circle centred on the optic disc with real-time artefact reduction technology set to 100 (axial protocol). The acquisition rate was 40 000 A-scans per second at an axial resolution of 3.9 µm. A volumetric (20 × 20^°^ volume) scan of the macula centred on the fovea was then performed (73 B-scans covering a superior-inferior distance of 4.6 mm). En face OCTA images were acquired with the Heidelberg Spectralis angiography. Macular images were taken from a 10^°^ × 10^°^ (c. 2.9 × 2.9 mm) scanning area centred on the fovea centralis with active eye tracking capabilities for the OCTA image.

OCT scans were quality checked using the OSCAR-IB criteria before extraction, which has been shown to improve inter-rater agreement of scan quality.^17^ Scans not meeting these criteria, or with low signal strength (<25), were excluded from analysis. Retinal layers were segmented with the inbuilt software (Eye Explorer v.11.2.0). The quality of automated segmentation was checked and manually corrected as necessary (CY, PS). The macular ganglion cell inner plexiform layer (GCIPL) and inner nuclear layer (INL) were calculated based on average thicknesses obtained from an Early Treatment Diabetic Retinopathy Study (*ETDRS*) circular *grid* map, encompassing a 6 × 6 mm cylinder centred on the fovea.

OCTA MIP images were excluded if the quality/signal score was <30 or did not meet the OSCAR-MP criteria.^18^ The manufacturer derived algorithm (Eye Explorer v.11.2.0) segmented the macular images into the superficial (SVC) and deep vascular complex (DVC) and segmentation checked and errors manually corrected as necessary (CY). For this analysis only the SVC was analysed.

**Figure 1:**
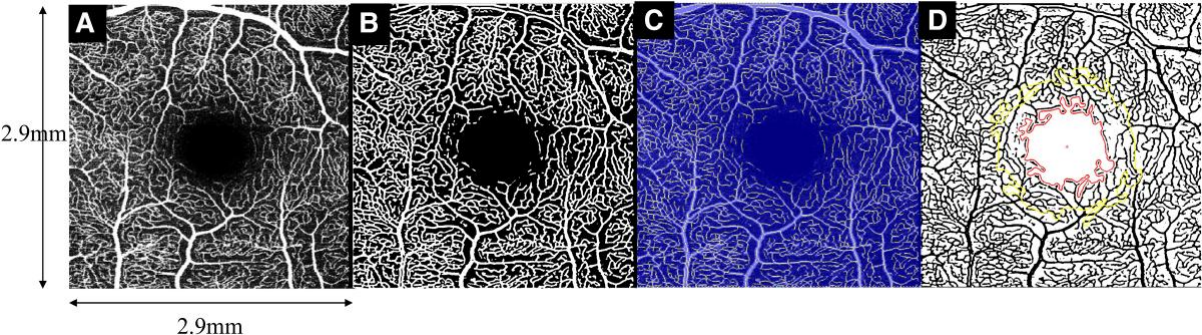
**Visualization of images used for generation of OCTA derived microvasculature metrics:** (**A**) Raw superficial vascular complex maximum intensity projection (MIP) image (**B**) Binarized image (**C**) Skeletonized image (**D**) Segmentation of the foveal avascular zone. Images are generated using the OCTAVA toolbox^19^.

OCTAVA toolbox (R2002B) was utilized to calculate the retinal vasculature metrics^19,20^ (Fig. 1). The region of interest used for analysis was the entire 3 × 3 mm square image centred on the fovea as the reference point. The foveal avascular zone segmentation was checked for accuracy and corrected manually if necessary (CY) and was vital for accurate measurement of the retinal microvasculature. Metrics of vascular drop-out generated through the OCTAVA toolbox included vessel area density (VAD) and vessel length density (VLD), which reflect the extent and distribution of blood vessels within the retina. Metrics reflective of changes in vessel architecture include FD, which describes the complexity of the retinal vascular network and reflects how the blood vessels branch out in the retina.

### Visual and clinical assessment

Best corrected, monocular vision was recorded in MS patients. High-contrast visual acuity (HCVA) was tested and measured with a 3-m LogMAR acuity chart to obtain LogMAR values. Low-contrast visual acuity (LCVA) was measured with a 2-m 2.5% contrast level LCVA charts displayed on an illuminated Precision Vision lightbox (230 Volts/50 Hz frequency) with the room light off and quantified as the number of correctly identified letters. Colour vision using Ishihara Plates and scored out of 16 (which excluded the test plate). Participants with congenital dyschromatopsia underwent all assessments but not colour vision assessment. Disability was assessed with EDSS^21^ by a trained assessor.

### Magnetic resonance imaging acquisition and analysis

MRI was performed in patients only. MRI data were acquired using a 3T Philips Ingenia CX system (Philips Healthcare, Best, The Netherlands) with a 32-channel head coil. High-resolution (1 mm^3^ isotropic) 3D T1-weighted images were acquired for brain volumetric measurements. A magnetization-prepared 3D Turbo Field Echo sequence was used with the following parameters: number of slices = 176, repetition time (TR) = 28 ms, echo time (TE) = 7 ms, flip angle (FA) = 24°, voxel size = 1.0 × 1.0 × 1.0 mm³, acquired in the sagittal orientation. In addition, 3D T2-weighted fluid-attenuated inversion recovery (FLAIR) data were acquired for each subject using the following parameters: voxel size = 1.2 mm³ isotropic, number of slices = 176, repetition time (TR) = 4800 ms, inversion time = 1650 ms, echo time (TE) = 268 ms, acquired in the sagittal orientation.

T2 FLAIR images were used for the segmentation of white matter lesions. Lesion detection was conducted using a single cascade version of NicMSlesion.^22^ This is a supervised machine learning based technique which implements a single convolutional neural network to classify lesion voxels. The model used for this segmentation has been trained using clinical trial data (MS-STAT2) acquired on the same MRI scanner and has been previously validated in house.^23^ Lesion masks were checked and manually corrected by trained raters.

For each participant, the FLAIR volume was then linearly co-registered to the 3D T1 using NiftyReg,^24,25^ and the obtained transformations were applied to the lesion masks to enable lesion filling using a patch-based technique.^26^

Anatomical T1-weighted lesion-filled images were used for whole brain tissue class segmentation. Whole brain images were segmented in native subject space into gross tissues classes (cortical grey matter, white matter, deep grey matter, cerebrospinal fluid), as well as parcellated regional brain volumes^27^ using Geodesical Information Flows segmentation software.

White and grey matter brain volumes were adjusted for total intracranial volume (TIV) in regression models. The residuals of these regression models were considered the new, *adjusted*, grey and white matter values to be used in subsequent steps.^28^ Regional cortical volumes were corrected for total grey matter (adjusted values). Visual pathway structures/cortices and non-visual cortices were examined (the latter as qualitative controls).

### Defining optic neuritis status

Eyes affected by optic neuritis were designated with the following criteria: thinner eye of a pair if inter-eye differences were ≥ 5 µm for pRNFL and ≥ 4 µm for mGCIPL.^29^ For remaining OCT metrics, if eyes showed an absolute pRNFL thickness <75 µm or mGCIPL thickness <52 µm (calculations based on <2.5 standard deviations below the weighted pRNFL and GCIPL mean for a healthy cohort aged between 18 and 70 + years, obtained from a Heidelberg Spectralis machine),^29,30^ these were additionally assigned as eyes affected by optic neuritis.

### Statistical analysis

Analyses were performed using R Studio (R 4.1.2) (Posit, Massachusetts, USA). Differences in demographic variables between HCs and MS patients were explored with two sample *t* tests or chi-squared tests of independence (for numeric or categorical).

Optic neuritis and eye status (HC, non-optic neuritis, optic neuritis) were initially assessed as predictors of OCT (pRNFL, mGCIPL) and superficial vascular plexus OCTA (VAD, VLD, FD) metrics, adjusting for age and sex at eye level in linear mixed models with participants as random intercepts to account for within-subject eye correlations.

Further analyses for MS patients were performed using linear mixed models with participants as random intercepts in two forms. (i) Visual outcomes (HCVA, LCVA, colour Vision) as response variables were assessed, in turn, with OCT/OCTA measures as predictors. (ii) The relationships between EDSS and MRI brain volumes and OCT/OCTA metrics were explored in linear mixed models, with EDSS and MRI brain volumes as predictors and OCT/OCTA as outcomes. Because patient-level outcomes (EDSS or MRI metrics) are not replicated across eyes, modelling eye-level visual outcomes as dependent variables with patient-level predictors allows valid estimation of associations while accounting for intra-subject correlation. As eye-based parameters were correlated with subject-based parameters, a random effect was included in the model to account for two eyes as non-independent observations nested within each subject. These models were adjusted for covariates age, sex, optic neuritis status, disease duration and proportion of disease duration on DMT as fixed effects.

Multicollinearity was assessed in the models, and an acceptable VIF (variance inflation factor) was considered as <5.^31^ Interaction analyses were conducted between EDSS and disease duration/optic neuritis status in predicting OCT/OCTA and between OCT/OCTA and optic neuritis/disease duration status in predicting vision outcomes and between MRI metrics and optic neuritis/disease duration status in predicting OCT/OCTA. The most suitable interaction term included in the final reported model was determined by the lowest Akaike Information Criteria (AICc) on model comparison.^32^

Post-hoc pairwise comparisons compared OCT/OCTA measures between disease groups (optic neuritis, non-optic neuritis, healthy control) applying the Tukey multiple comparisons adjustment. Vision outcome models were compared (non-nested) using the corrected AICc and AICc weight (AICcWt).^32^ We applied a two-sided significance level of <0.01 rather than 0.05, as multiple models were evaluated in this study.

ROC analyses evaluated OCT and OCTA metrics for discriminating non-optic neuritis eyes from healthy controls, with area under the curves (AUCs) reported as a Supplementary analysis.

## Results

**Figure 2:**
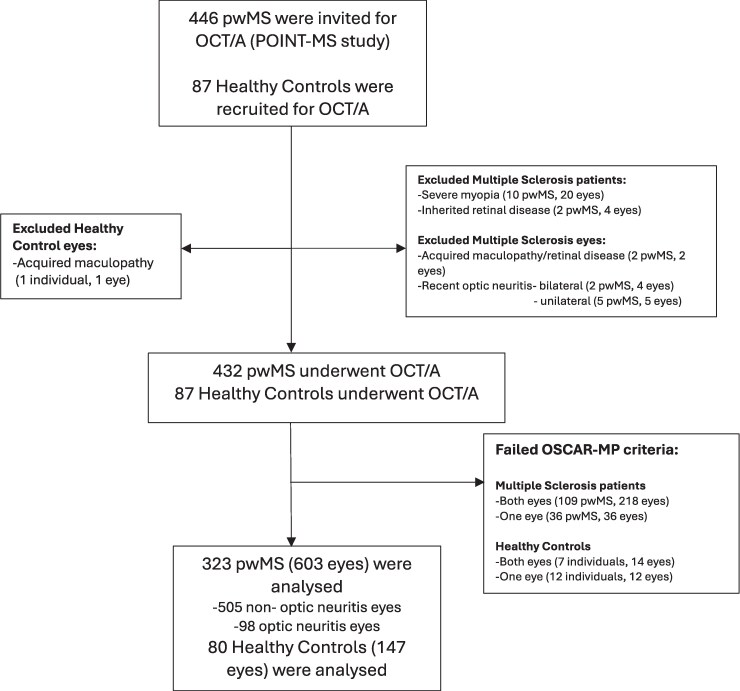
**Flow-chart of final patients and eyes analysed after exclusion criteria.** Abbreviations: pwMS: people with Multiple Sclerosis, OCT/A: Optical coherence tomography and angiography, POINT-MS study: Predicting Optimal Individualized Treatment Response in Multiple Sclerosis study. OSCAR-MP criteria is a 7-component quality control criteria including Obvious problems, Signal Strength, Centration, Algorithm failure, Retinal pathology, Motion artefacts and Projection artefacts.

Out of 432 MS patients and 87 controls who underwent OCT/OCTA, 323 patients (603 eyes, 98 with previous optic neuritis) and 80 healthy controls (147 eyes) were analysed (see flow chart in Fig. 2). The demographic and clinical characteristics of the patients and healthy controls are shown in Table 1. A total of 184 patients (57%) were starting their first DMT at enrolment into the POINT-MS study. The details of the DMT switches for the remaining non-naïve patients are shown in Supplementary Table 1.

**Table 1:** Summary of demographic and clinical characteristics of the multiple sclerosis and healthy control cohorts

		
Summary	*Multiple sclerosis (n = 323)*	*Healthy controls (n = 80)*
**Age (years)** ^a^	39.92 (10.37)	37.90 (11.40)
**Sex (female)**	228 (71%)	48 (60%)
**Cardiovascular comorbidities** ^b^	19 (5.90%)	18 (22.50%)
Current smokers	20 (6.20%)	6 (7.50%)
Body mass index^a^	24.03 (21.60–27.50)	-
**Disease duration (years)** ^a^	6.13 (2.35–11.26)	-
**MS phenotype**		
Relapsing remitting	314 (97.20%)	
Primary progressive	8 (2.50%)	
Secondary progressive	1 (0.30%)	
**EDSS (median, IQR)** ^a^	2.0 (1.0–3.0)	
**Ethnicity**		
White participants	252 (78.02%)	
Non-white participants	71 (21.98%)	
**Current DMT**		
Ocrelizumab^c^	147 (45.5%)	
Ofatumumab^c^	129 (40%)	
Cladribine^d^	20 (6.2%)	
Dimethyl fumarate^d^	5 (1.55%)	
Fingolimod^d^	1 (0.3%)	
Glatiramer acetate^d^	12 (3.7%)	
Interferon beta^d^	5 (1.55%)	
Natalizumab^c^	1 (0.3%)	
Teriflunomide^d^	1 (0.3%)	
Siponimod^d^	1 (0.3%)	
Alemtuzumab^c^	1 (0.3%)	
**Proportion of disease duration on DMT** ^a^	0.26 (0.043–0.96)	
**Time on current DMT (years)** ^a^	0.13 (0.0055–0.84)	
MRI metrics	*MS patients (n = 267)*	
Total intracranial volume (ml)^a^	1447 (135.60)	
T2 lesion volume (ml)^a^	6.40 (3.02–12.70)	
Number of lesions^a^	37.5 (24–57)	
White matter (ml)^a^	443 (50.80)	
Cortical grey matter (ml)^a^	600.40 (55.00)	
Deep grey matter (ml)^a^	35.30 (3.80)	
Thalamus (ml)^a^	11.10 (1.30)	

Vision metrics (median, IQR)	*MS eyes (n = 603)*	*non-ON eyes (n = 505)*	*ON eyes (n = 98)*
LogMAR (best corrected)^a^	−0.1 (−0.2–0.0)	−0.1 (−0.2–0.0)	−0.1 (−0.1–0.0)
Sloan 2.5%^a^	31 (24–35)	32 (25–36)	21.5 (6.25–30.75)
Ishihara Plates (score out of 16)^a^	16 (15–16)	16 (15–16)	15 (13–16)

MS = multiple sclerosis.

^a^Summary data are expressed mean (standard deviation, SD) or median (interquartile range, IQR) depending on the distribution of the data.

^b^Cardiovascular comorbidities include hypertension, diabetes mellitus and dyslipidaemia.

^c^High-efficacy DMTs: Ocrelizumab, Ofatumumab, Natalizumab, Alemtuzumab.

^d^Non-high-efficacy DMTs: Interferon beta, Dimethyl fumarate, Glatiramer acetate, Teriflunomide, Fingolimod, Siponimod, Cladribine.

The two groups were similar in age (*t* = −1.525, df = 401, *P* = 0.13), sex (χ^2^ (1, *n* = 403) = 3.33, *P* = 0.07) and smoking status (χ^2^ (3, *n* = 403) = 0.96, *P* = 0.81), but a higher proportion of healthy controls had cardiovascular comorbidities (including hypertension, diabetes mellitus or dyslipidaemia) than patients (χ^2^ (1, *n* = 403) = 12.48, *P* < 0.001) (Table 1). Generally, the prevalence of comorbidities in the patient group was low (5.9%).

Most patients (97.2%) were relapsing remitting, with mild EDSS (median 2, range 1–3); a minority of patients self-identified as Black (4.64%), Asian (9.3%) or Other Ethnicities (8.04%), and the remaining 78.02% as White. Most patients (86.1%) started a high-efficacy therapy. The MS cohort as a group had preserved visual acuity and colour vision, especially in non-optic neuritis eyes (Table 1).

### Optical coherence tomography and optical coherence tomography angiography metrics in multiple sclerosis patients and healthy controls

**Figure 3:**
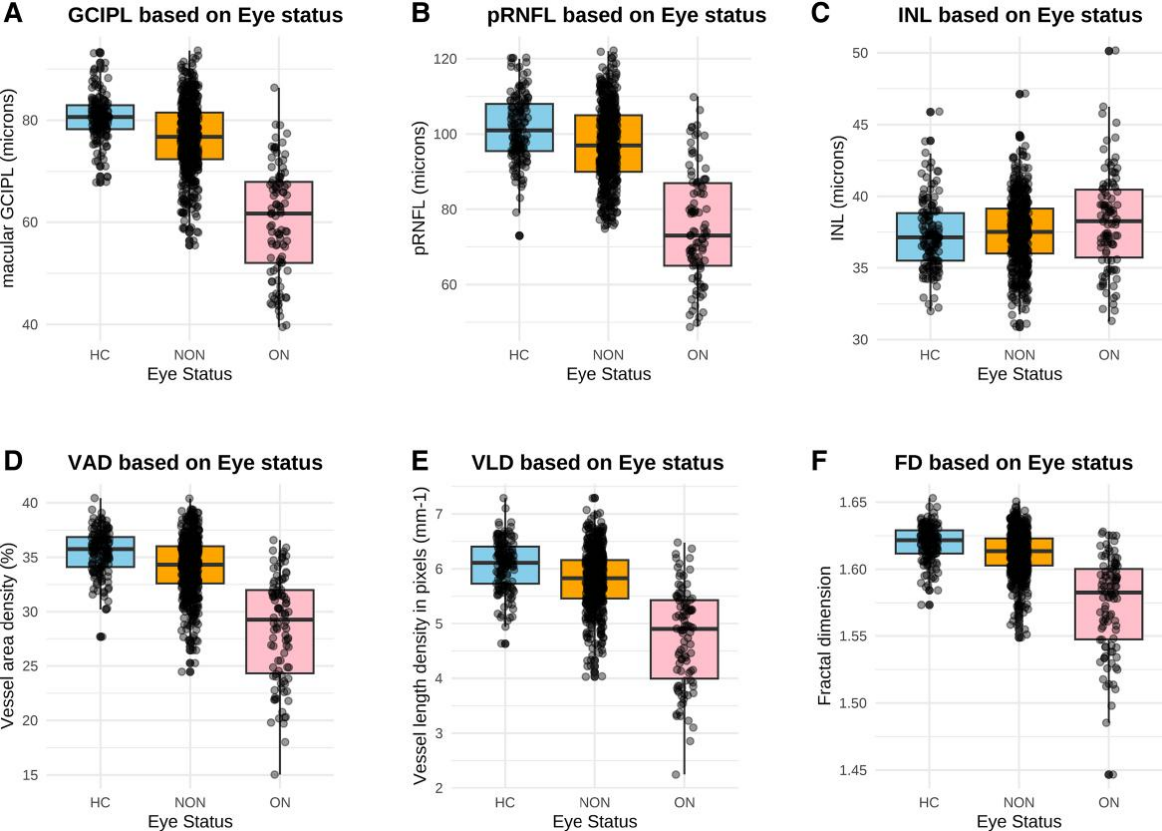
**OCTA metrics in healthy controls (HC) versus non-optic neuritis (NON) versus optic neuritis (ON) eyes.** Box plots showing summary statistics [boxes representing the interquartile range (25th–75th percentile) and the line inside the box representing the median] for healthy control (blue), non-optic neuritis (orange) and optic neuritis eyes (pink) for (**A**) mGCIPL thickness (**B**) pRNFL thickness (**C**) INL thickness (**D**) Vessel area density (**E**) Vessel length density and (**F**) fractal dimension. No statistical comparisons were performed. Sample sizes: Healthy controls (*n* = 147 eyes), non-optic neuritis (*n* = 505 eyes) and optic neuritis (*n* = 98 eyes). Each datapoint represents a measure taken from an individual eye per patient.

Table 1 and Fig. 3 summarizes unadjusted OCT/OCTA metrics at eye level. From the linear mixed models, VAD, VLD and FD were significantly reduced in non-optic neuritis eyes of patients compared with healthy controls (estimated marginal mean differences: VAD [−1.28%], VLD [−0.27 pixels mm^−1^], FD [−0.0081], *P* < 0.001) and, in patients, between optic neuritis eyes and non-optic neuritis eyes (estimated marginal mean differences: VAD [−4.63%], VLD [−0.87 pixels mm^−1^], FD [−0.031], *P* < 0.0001) (Supplementary Table 2).

Similarly, mGCIPL and pRNFL thicknesses were reduced in non-optic neuritis eyes compared with control eyes (estimated marginal mean differences: mGCIPL [−4.44 μm], pRNFL [−5.26 μm], *P* < 0.001), and in optic neuritis eyes compared with non-optic neuritis eyes (estimated marginal mean differences: mGCIPL [−12.6 μm], pRNFL [−15.42 μm], *P* < 0.0001). There was also significantly increased mINL thickness in optic neuritis eyes compared with non-optic neuritis eyes (estimated marginal mean difference of +0.80 μm, *P* < 0.0001) but no significant difference in this metric between non-optic neuritis eyes and healthy controls (Fig. 3 and Supplementary Table 2).

### Associations between MRI parameters and optical coherence tomography angiography/optical coherence tomography metrics

267 patients had MRI brain performed within 90 days of OCT/OCTA. Another three patients with MRI scans > 90 days from OCT/A were excluded. These 267 patients contributed 496 eyes (74 with optic neuritis) for analysis in the MRI models. The summary brain volume statistics are shown in Table 1.

In patients, decreased OCTA (VAD and VLD) and OCT (mGCIPL) were significantly associated with reduced cortical grey matter volumes (all *P* < 0.01), but not pRNFL (*P* = 0.086) (Table 2). Thinner mGCIPL was also associated with higher T2 lesion volumes (*P* = 0.0047) and lesion numbers (*P* = 0.0025). In contrast, thinner pRNFL was uniquely associated with reducing white matter volumes (*P* = 0.008) and borderline significantly associated with reducing deep grey matter volumes (*P* = 0.043). OCTA and pRNFL were not associated with T2 lesion volume or number (*P* > 0.01) (Table 2).

**Table 2:** Linear mixed models exploring the relationship between global MRI metrics and OCT/A

OCT/A metric	b coefficient	Standard error	95% CI	*P*-value
***Vessel area density (VAD)***				
T2 lesion volume^a^	−0.050	0.022	−0.094 to −0.0068	0.023
White matter volume^a,b^	0.020	0.011	−0.0057 to 0.037	0.15
Deep grey matter volume^a,b^	0.050	0.089	−0.12 to 0.23	0.54
Cortical grey matter volume^a,b^	0.040	0.014	0.012 to 0.067	0.0055*
Lesion number	−0.015	0.0062	−0.027 to −0.0024	0.019
***Vessel length density (VLD)***				
T2 lesion volume^a^	−0.0090	0.0043	−0.018 to −0.0011	0.029
White matter volume^a,b^	0.0020	0.0021	−0.0022 to 0.0062	0.34
Deep grey matter volume^a,b^	0.0050	0.017	−0.029 to 0.04	0.76
Cortical grey matter volume^a,b^	0.0080	0.0028	0.0023 to 0.013	0.0053*
Lesion number	−0.0026	0.0012	−0.0050 to −0.00022	0.033
***Fractal dimension (FD)***				
T2 lesion volume^a^	−0.00020	0.00015	−5.18e−04 to 5.24e−05	0.11
White matter volume^a,b^	0.00010	0.000072	−8.06e−05 to 2.01e−04	0.40
Deep grey matter volume^a,b^	0.00030	0.00056	−7.94e−04 to 1.41e−03	0.58
Cortical grey matter volume^a,b^	0.00020	0.000093	3.66e−05 to 4.02e−04	0.019
Lesion number	−0.000072	0.000040	−0.00015 to 7.57e−06	0.076
***mGCIPL***				
T2 lesion volume^a^	−0.16	0.056	−0.27 to −0.049	**0.0047** *
White matter volume^a,b^	0.050	0.028	−0.0064 to 0.10	0.083
Deep grey matter volume^a,b^	0.38	0.23	−0.065 to 0.83	0.093
Cortical grey matter volume^a,b^	0.090	0.036	0.024 to 0.17	**0.0088** *
Lesion number	−0.048	0.016	−0.079 to −0.017	0.0025*
***pRNFL***				
T2 lesion volume^a^	−0.14	0.079	−0.29 to 0.018	0.082
White matter volume^a,b^	0.10	0.039	0.027 to 0.18	**0.0080** *
Deep grey matter volume^a,b^	0.65	0.32	0.020 to 1.27	0.043
Cortical grey matter volume^a,b^	0.090	0.051	−0.013 to 0.19	0.086
Lesion number	−0.014	0.023	−0.059 to 0.031	0.54

Baseline effects (reference categories and intercepts) reported. All models corrected for optic neuritis status (interaction factor), age, sex, disease duration and proportion of disease duration on DMT.

^a^Metrics based on brain volumes/T2 lesion volume in millilitres.

^b^White and deep grey matter volumes corrected for TIV.

^*^Statistical significance defined as ≤0.01 and shown in **bold**.

For regional brain volume associations, we assessed both visual-related regions and non-visual related regions (within the frontal, parietal and temporal lobes) as qualitative controls.

Generally, OCTA (Table 3) and OCT metrics (Table 4) showed significant associations with visual-related cortical regions, but not with frontal, non-visual temporal or parietal cortical regions (Supplementary Table 3).

**Table 3:** Linear mixed models exploring the relationship between regional visual pathway structure volumes and OCTA

OCTA metric	b coefficient	Standard error	95% CI	*P*-value
***Vessel area density (VAD)***				
***(i) Anterior pathway***				
Optic chiasm	25.74	17.94	−9.60 to 61.07	0.15
Thalamus^a^	0.52	0.14	0.24 to 0.81	0.00040*
***(ii) Primary visual cortex*** ^a^				
Occipital pole	0.66	0.23	0.21 to 1.11	0.0040*
Calcarine cortex	0.50	0.14	0.21 to 0.78	0.00070*
***(iii) Visual association cortices*** ^a^				
Superior occipital gyrus	0.44	0.19	0.065 to 0.81	0.022
Inferior occipital gyrus	0.28	0.11	0.066 to 0.49	0.010*
Cuneus	0.36	0.13	0.096 to 0.62	0.0077*
Occipital fusiform gyrus	0.51	0.18	0.17 to 0.86	0.0039*
Lingual gyrus	0.25	0.098	0.054 to 0.44	0.01
Inferior temporal gyrus	0.22	0.087	0.046 to 0.39	0.013
Superior parietal gyrus	0.26	0.10	0.059 to 0.46	0.012
***Vessel length density (VLD)***				
***(i) Anterior pathway***				
Optic chiasm	4.64	3.51	−2.28 to 11.56	0.19
Thalamus^a^	0.10	0.028	0.046 to 0.16	0.00040*
***(ii) Primary visual cortex*** ^a^				
Occipital pole	0.13	0.045	0.042 to 0.22	0.0039*
Calcarine cortex	0.092	0.028	0.036 to 0.15	0.0012*
***(iii) Visual association cortices*** ^a^				
Superior occipital gyrus	0.077	0.037	0.0034 to 0.15	0.040
Inferior occipital gyrus	0.057	0.021	0.016 to 0.099	0.0069*
Cuneus	0.070	0.026	0.018 to 0.12	0.0078*
Occipital fusiform gyrus	0.094	0.034	0.026 to 0.16	0.0068*
Lingual gyrus	0.045	0.019	0.0075 to 0.083	0.019
Inferior temporal gyrus	0.044	0.017	0.011 to 0.078	0.0093*
Superior parietal gyrus	0.053	0.020	0.013 to 0.092	0.0089*
***Fractal dimension (FD)***				
***(i) Anterior pathway***				
Optic chiasm	0.15	0.12	−0.081 to 0.39	0.20
Thalamus^a^	0.0030	0.00096	0.0011 to 0.0049	0.0018*
***(ii) Primary visual cortex*** ^a^				
Occipital pole	0.0039	0.0015	0.00094 to 0.0068	0.010*
Calcarine cortex	0.0028	0.00095	9.05e−04 to 4.64e−03	0.0038*
***(iii) Visual association cortices*** ^a^				
Superior occipital gyrus	0.0023	0.0013	−1.20e−04 to 4.8e−03	0.062
Inferior occipital gyrus	0.0018	0.00071	4.13e−04 to 3.22e−03	0.011
Cuneus	0.0021	0.00087	3.80e−04 to 3.82e−03	0.017
Occipital fusiform gyrus	0.0030	0.0011	7.55e−04 to 5.31e−03	0.0092*
Lingual gyrus	0.0014	0.00065	1.46e−04 to 2.69e−03	0.029
Inferior temporal gyrus	0.0013	0.00057	0.00020 to 0.0024	0.020
Superior parietal gyrus	0.0016	0.00068	0.00027 to 0.0029	0.019

Baseline effects (reference categories and intercepts) reported. All models corrected for ON status (interaction factor), age, sex, disease duration and proportion of disease duration on DMT. *Statistical significance defined as ≤0.01 and shown in **bold**.

^a^Parcellated volumes corrected for grey matter volume, except for optic chiasm.

**Table 4:** Linear mixed models exploring the relationship between regional visual pathway structure volumes and OCT

OCT metric	b coefficient	Standard error	95% CI	*P*-value
***mGCIPL***				
***(i) Anterior pathway***				
Optic chiasm	111.82	45.1	23.0 to 200.59	0.014
Thalamus^a^	1.31	0.37	0.58 to 2.03	0.0005*
***(ii) Primary visual cortex*** ^a^				
Occipital pole	1.16	0.59	0.0048 to 2.32	0.049
Calcarine cortex	1.05	0.37	0.32 to 1.78	0.0049*
***(iii) Visual association cortices*** ^a^				
Superior occipital gyrus	1.30	0.49	0.34 to 2.25	0.0079*
Inferior occipital gyrus	0.31	0.28	−0.24 to 0.86	0.27
Cuneus	0.55	0.34	−0.12 to 1.23	0.11
Occipital fusiform gyrus	0.46	0.45	−0.43 to 1.35	0.31
Lingual gyrus	0.40	0.25	−0.098 to 0.90	0.11
Inferior temporal gyrus	0.45	0.22	0.007 to 0.89	0.047
Superior parietal gyrus	0.75	0.26	0.23 to 1.26	0.0046*
***pRNFL***				
***(i) Anterior pathway***				
Optic chiasm	293.30	61.60	171.98 to 414.63	<0.0001*
Thalamus^a^	2.21	0.52	1.18 to 3.24	<0.0001*
***(ii) Primary visual cortex*** ^a^				
Occipital pole	2.65	0.83	1.02 to 4.29	0.0016*
Calcarine cortex	2.60	0.52	1.58 to 3.61	<0.0001*
***(iii) Visual association cortices*** ^a^				
Superior occipital gyrus	2.95	0.68	1.62 to 4.28	<0.0001*
Inferior occipital gyrus	0.55	0.40	−0.24 to 1.33	0.17
Cuneus	1.30	0.49	0.35 to 2.26	0.0078*
Occipital fusiform gyrus	1.07	0.64	−0.20 to 2.34	0.099
Lingual gyrus	0.76	0.36	0.047 to 1.47	0.037
Inferior temporal gyrus	0.64	0.32	0.011 to 1.26	0.046
Superior parietal gyrus	0.94	0.37	0.21 to 1.68	0.012

Baseline effects (reference categories and intercepts) reported. All models corrected for ON status (interaction factor), age, sex, disease duration and proportion of disease duration on DMT. *Statistical significance defined as ≤0.01 and shown in **bold**.

^a^Parcellated volumes corrected for grey matter volume, except for optic chiasm.

Regarding visual-related regions (Tables 3 and 4), reduced OCTA metrics; VAD and especially VLD, revealed more significant associations with higher-order visual areas (e.g. cuneus, occipital fusiform gyrus) than did FD and OCT metrics. In particular, VLD exhibited significant associations with five out of seven higher visual areas [inferior occipital gyrus (*P* = 0.0069), cuneus (*P* = 0.0078), occipital fusiform gyrus (*P* = 0.0068), inferior temporal gyrus (*P* = 0.0093) and superior parietal (*P* = 0.0089) gyrus], whereas VAD was associated with three higher visual regions (inferior occipital gyrus, cuneus, occipital fusiform gyrus), mGCIPL and pRNFL with two each (mGCIPL: superior occipital and parietal gyrus, pRNFL: superior occipital gyrus and cuneus), and FD with one (occipital fusiform gyrus) (all *P* ≤ 0.01) (see Table 3). Regarding primary visual cortices, optic chiasm and thalamus, there was greater overlap between OCTA and OCT metrics for significant associations with thalamus and calcarine cortex being consistently associated with VAD, VLD, mGCIPL and pRNFL. For frontal, parietal and temporal regions considered non-visual related, no significant associations were seen for OCTA/OCT metrics (Supplementary Table 3).

### Associations between EDSS and optical coherence tomography angiography metrics

A total of 323 patients had EDSS assessments; significant associations were seen between OCT/OCTA metrics and EDSS, except for pRNFL (Table 5).

**Table 5:** Linear mixed models exploring the relationship between visual scores, EDSS and OCT/A

Clinical outcome	b coefficient	Standard error	95% CI	*P*-value
***Logmar*** ^a^				
VAD	−0.0096	0.0021	−0.014 to −0.0055	<0.0001*
VLD	−0.042	0.010	−0.062 to −0.022	<0.0001*
FD	−1.43	0.33	−2.09 to −0.78	<0.0001*
mGCIPL	−0.0021	0.00081	−0.0037 to −0.00051	0.010*
pRNFL	−0.00092	0.00060	−0.0021 to 0.00026	0.12
***Sloan 2.5% LCVA*** ^a^				
VAD	1.01	0.15	0.70 to 1.31	<0.0001*
VLD	4.87	0.73	3.43 to 6.31	<0.0001*
FD	152.0	23.94	104.86 to 199.14	<0.0001*
mGCIPL	0.40	0.058	0.29 to 0.51	<0.0001*
pRNFL	0.19	0.043	0.11 to 0.28	<0.0001*
***Ishihara colour vision*** ^a^				
VAD	0.084	0.029	0.028 to 0.14	0.0040*
VLD	0.38	0.14	0.099 to 0.65	0.0080*
FD	12.43	4.37	3.82 to 21.04	0.0050*
mGCIPL	0.034	0.011	0.011 to 0.057	0.0030*
pRNFL	0.020	0.0087	0.0025 to 0.037	0.03
***EDSS*** ^b^				
VAD	−0.51	0.15	−0.81 to −0.21	0.0010*
VLD	−0.091	0.030	−0.15 to −0.031	0.0028*
FD	−0.0028	0.0010	−0.0048 to −0.00081	0.0059*
mGCIPL	−1.52	0.40	−2.3 to −0.75	0.0001*
pRNFL	−1.37	0.56	−2.48 to −0.27	0.015

Baseline effects (reference categories and intercepts) reported.

^a^Models corrected for age, gender, disease duration and proportion of disease on DMT and optic neuritis status (as an interaction term).

^b^Models corrected for age, sex, proportion of disease on DMT, optic neuritis status and disease duration (as an interaction term).

^*^Statistical significance defined as ≤0.01 and shown in **bold**.

From interaction analyses, at shorter disease duration there was a significant association between EDSS and OCTA/OCT but not for longer disease duration (See Supplementary Fig. 1). For every additional year of disease duration, the association between EDSS and OCTA/OCT became less negative by the following steps: slope increased by 0.036% for VAD (95% CI = 0.013–0.059, *P* = 0.0018), 0.0067 pixels/mm for VLD (95% CI = 0.0023–0.0067, *P* = 0.0031), 0.00021 units for FD (95% CI = 0.000065–0.00036, *P* = 0.005), 0.092 µm for mGCIPL (95% CI = 0.034–0.15, *P* = 0.002) and 0.11 µm for pRNFL (95% CI = 0.025–0.19, *P* = 0.011), per EDSS point.

### Associations between visual scores and optical coherence tomography angiography metrics

Unadjusted, eye-level, summary statistics for visual outcomes are shown in Table 1. 321 patients underwent vision testing. A total of 296 patients had colour vision performed following exclusion for congenital colour blindness (*n* = 6 patients) and unavailability of the test.

The associations between OCT/A metrics and vision scores, from the linear mixed models, are shown in Table 5. Reduced VAD, VLD, FD were associated with worse vision (higher LogMAR scores, lower Sloan 2.5% low-contrast visual acuity scores, and lower Ishihara colour vision plates). Similarly for OCT, both reduced mGCIPL and pRNFL were associated with worse Sloan 2.5% low contrast visual acuity but only thinner mGCIPL was additionally associated with higher LogMAR scores and worse colour vision.

Generally, the slopes of associations between OCT/OCTA metrics with all visual outcomes were steeper for optic-neuritis eyes compared with non-optic neuritis eyes (Supplementary Fig. 2).

The models estimating OCT and OCTA relationships with each visual outcome were compared using corrected AICc. For Logmar, VAD was determined as the best model, followed by GCIPL, VLD and FD. For Sloan 2.5% LCVA, mGCIPL was the strongest model, followed by VAD, VLD and FD, which were all stronger than pRNFL. For Ishihara Plate colour testing, FD model was the best predictor, followed by VAD and VLD and then mGCIPL and all were stronger predictors than pRNFL.

### Supplementary analysis

- ROC curve analysis confirmed moderate discriminatory ability of OCT and OCTA metrics for distinguishing non-optic neuritis eyes from healthy controls, with AUC values ranging from 0.626 to 0.673 (Supplementary Fig. 3).
- Sensitivity analyses including high-efficacy DMT (HE-DMT) as a covariate instead of proportion of disease duration on DMT did not substantially change the model results (Supplementary Tables 4 and 5).
- Model comparisons using AICc indicated that interactions with optic neuritis status generally provided slightly better fit than interactions with disease duration for VAD and GCIPL across multiple MRI regions (Supplementary Table 6).

## Discussion

This study investigated retinal microvasculature as a potential biomarker reflecting MRI markers of inflammation and brain volume alterations in MS. It was conducted on a large cohort of predominantly relapsing patients and examined both optic neuritis and non-optic neuritis eyes. We compared associations found with OCTA against conventional OCT metrics to help understand cerebral damage mechanisms and clinical outcomes in MS and ascertain any complementary value of OCTA over OCT as a biomarker.

### Retinal microvasculature changes in multiple sclerosis compared with healthy controls

We observed abnormal retinal microvasculature in our POINT-MS cohort compared to age- and sex-matched healthy controls, as well as between optic neuritis and non-optic neuritis eyes, reinforcing previous studies that have reported reduced vessel density and altered vascular architecture in MS, independent of optic neuritis.^9,11,33^ Intrathecal immunity research suggests that retinal microvasculature rarefication correlates with increased activated B cells and pro-inflammatory cytokines in cerebrospinal fluid, indicating OCTA’s potential utility in detecting in vivo neuroinflammation.^34^ Moreover, previous studies have demonstrated distinct retinal vessel loss patterns between optic neuritis and non-optic neuritis eyes. In optic neuritis eyes, medium-sized retinal vessel reduction correlates with ganglion cell loss, suggesting altered retinal metabolism.^35^ Conversely, in non-optic neuritis eyes, there is loss of small vessels, which appears unrelated to ganglion cell degeneration, pointing to a compromised blood–retina barrier and inflammatory effects.^35^ Furthermore, a post-mortem study by Green *et al*. demonstrated abnormal distribution of tight junction proteins in some retinal vessels and an accumulation of HLA-DR–positive immune cells in the retinal layers and optic nerve head near vessels.^36^ These pathological findings complement *in vivo* evidence, supporting a role for compartmentalized inflammation and vascular barrier dysfunction in MS-related retinal changes. Retrograde degeneration is also likely to contribute, as previous studies have shown effects on pRNFL/GCIPL thinning in non-optic neuritis eyes^37^ although its impact on the retinal microvasculature remains less well defined.

### Brain volume associations differ between optical coherence tomography angiography and optical coherence tomography metrics

Our findings reveal differential correlations between global brain volumes, primary visual pathway regions, and higher order visual structures with OCTA and OCT metrics. OCTA and mGCIPL thinning were reflective of cortical grey matter atrophy, whereas pRNFL thinning was associated with white matter atrophy and borderline significant correlations with deep grey matter atrophy. This may suggest different mechanisms at play; cortical neurodegeneration may stem from chronic hypoperfusion leading to retrograde degeneration and mGCIPL thinning^38^ as well as an increased energy requirements in grey matter compared to white matter leading to relative hypoxia,^39^ whilst pRNFL changes may be linked to axonal loss involving deep grey matter connections.^40^

Previous imaging studies support this hypothesis, demonstrating reduced cortical microvascular oxygenation and grey matter hypoperfusion in MS.^8,41^ These patterns also align with experimental models showing that early hypoxia precede clinical symptoms.^42^

We found no correlation between OCTA metrics with lesion volume and number, which is in keeping with previous smaller clinically isolated syndrome and relapsing remitting MS cohort studies suggesting that OCTA reflects neurodegeneration from chronic hypoperfusion rather than acute inflammation in demyelinating lesions.^12^

### Retinal microvascular dysfunction reflects a network of atrophic changes along the visual pathway

Further analysis identified an association between regional cortical grey matter atrophy and worse OCTA metrics, which possessed a predilection for higher-order visual cortices while OCT metrics were more strongly linked to early visual pathway structures.

- **Primary visual cortex and visual association cortices:** All OCTA and OCT metrics were associated with smaller volumes of the primary visual cortex, but OCTA uniquely reflected alterations in higher-order visual processing regions (occipital or non-occipital lobe), such as the cuneus, inferior occipital, occipital fusiform and inferior temporal gyri.^14,43^
- **Thalamus:** Associations were found with all OCTA and OCT metrics and smaller thalamic volume, likely due to its vulnerability to disrupted connectivity, relay-centre function and subjectivity to both anterograde and retrograde degeneration at the synapse, as well as high metabolic activity and microglial density.^44-47^
- **Anterior visual pathway:** pRNFL thinning was uniquely associated with chiasm volume loss, reinforcing the link between Wallerian degeneration and white matter atrophy in visual pathway degeneration.

This suggests that the vascular alterations detected by OCTA may extend beyond early visual pathway degeneration, and is potentially linked to global hypoperfusion, increased metabolic demand, and possible susceptibility of posterior circulation structures to ischaemia.^48-51^ These mechanisms are likely all contributing and interacting as we found that non-visual cortical regions in the frontal, parietal and temporal lobes were not associated with OCTA or OCT, suggesting that global hypoperfusion is not the sole mechanism. Thus, the global cortical grey matter volume association with OCTA is likely driven by visual and extra-visual regions. Although a posterior-anterior gradient of atrophy was plausible, this could not be disentangled easily from functional visual cortical involvement, and the spatial distribution of associations suggests a predominance in visual and adjacent posterior regions.

An alternative explanation for the observed OCTA–brain volume associations is that they may partly reflect retrograde neurodegeneration or diminished metabolic and energy requirements of dysfunctional neurons along the visual pathway, leading to secondary retinal vascular and structural changes. Such mechanisms have been described in other neurodegenerative contexts^37^ and could contribute to the OCTA alterations we observed. However, the distinct topographical association patterns between OCT and OCTA in our cohort argue against retrograde degeneration as the sole explanation. OCT measures, particularly pRNFL, were most strongly related to anterior visual and white matter structures, whereas OCTA metrics were more consistently linked to higher-order visual cortices. This suggests that OCT and OCTA may capture complementary aspects of neuroaxonal and vascular–metabolic integrity. Longitudinal or multimodal imaging studies, including perfusion or connectivity analyses, will be needed to disentangle these processes and clarify causal relationships.

### Optical coherence tomography angiography reflects visual dysfunction and correlates with early physical disability in multiple sclerosis

Our study found strong correlations between altered OCT/OCTA metrics and poorer visual function, particularly in optic neuritis eyes. OCTA generally provided superior predictive models for high-contrast acuity and Ishihara colour vision, while mGCIPL was most predictive of Sloan low-contrast visual acuity. Interestingly, pRNFL proved to be a weaker predictor overall. Previous OCTA studies explored HCVA and LCVA associations,^11^ although associations with colour vision had not previously been examined. Colour vision loss, previously shown to have prognostic value in MS,^52^ was found to be more closely associated with OCTA than OCT. The association between OCTA metrics in non-optic neuritis eyes and colour processing centres in the occipital lobe (occipital fusiform and borderline significant for lingual gyrus) suggests cerebral hypoxia as one potential underlying mechanism.^53^ Importantly, these associations are observed even in an MS population with normal visual acuity and colour vision, indicating that such microvascular changes may occur early, before overt visual deficits are detectable. Although viewed as a screening tool, Ishihara plate testing can be administered quickly, and its value has been extended to predicting visual outcomes and axonal loss following ON.^54^ The unique associations between altered colour vision and retinal perfusion with respect to neuroimaging correlates expands our knowledge on the pathophysiological mechanisms of visual loss in MS.

Our findings support previous studies demonstrating that lower vascular density in the superficial plexus is associated with higher physical disability (EDSS scores),^11,55^ indicating a link between retinal microvascular abnormalities and physical disability in MS. Notably, these associations were more pronounced in MS with a disease duration of less than 10 years, suggesting that retinal ischaemia may be more relevant in the early stages of MS but less predictive of disability in later stages. Previous research on cerebral hypoxia has shown that hypoxia and hypoperfusion are linked to early lesion formation in MS,^56,57^ raising the possibility of a ‘window of opportunity’ for intervention. Addressing anoxic conditions in early disease could be an important consideration for neuroprotective strategies aimed at preventing progression. Further investigation in a larger cohort of progressive MS patients is warranted to clarify this hypothesis of early neurovascular susceptibility and to assess potential therapeutic implications.

### Heterogeneity among optical coherence tomography angiography metrics

The three OCTA metrics vessel area density (VAD), vessel length density (VLD) and FD capture related but distinct aspects of the retinal microvasculature. VAD quantifies the total area of perfused vessels, VLD represents the linear extent of the vasculature irrespective of vessel calibre and FD reflects the complexity of vascular branching. Weaker MRI volumetric associations seen with FD than vessel density may reflect its sensitivity to microvascular architecture rather than perfusion volume, so may be less sensitive to regional changes in brain atrophy, especially if compromised vascular supply is the main mechanism. For visual metrics, FD provided the best model fit for Ishihara colour vision, suggesting that early retinal changes in branching complexity in the retina, which could impact foveal cone integrity and metabolism could contribute in part to colour vision loss.^58^ Given the high metabolic demand of cone photoreceptors and the reliance of colour vision on intact foveal function, FD may better reflect subtle vascular insufficiency or early capillary pruning not yet mirrored in structural brain changes. In contrast, VAD was the strongest predictor of high-contrast visual acuity (LogMAR). This dissociation likely reflects the distinct physiological substrates underlying these visual functions. LogMAR acuity depends more on the overall integrity of the retina, which in turn relies on the total extent of vascular supply, effectively captured by VAD.^59^ VLD likely captures both the total length or extent of the vessels and vascular network complexity and may more reflect the biological processes of brain atrophy as a result. These findings suggest that different OCTA metrics capture complementary aspects of vascular integrity and may offer distinct structural and functional insights into visual pathway compromise in MS.

### Limitations

Several limitations should be considered when interpreting our findings. First, our study did not include an analysis of deeper retinal layers, particularly the DVC. While previous studies have reported limited significant results from DVC as a MS biomarker, the most impactful findings have predominantly come from the SVC.^11,12,35^ Future studies incorporating a broader retinal layer analysis could provide further insight into the role of microvascular changes in MS pathology. Second, approximately 25% of OCTA scans were excluded due to failure to meet OSCAR-MP quality control criteria. While rigorous QC ensures reliability of the data analysed, this exclusion rate reduced the final sample size and may limit the generalisability of findings. Future improvements in instrument imaging protocols or artefact correction may help mitigate such losses. Furthermore, potential selection bias cannot be excluded given that a proportion of eyes failed OCT-A quality control, most commonly from motion artefacts. Although the OCT-A subgroup had a comparable median EDSS to the overall POINT-MS cohort,^15^ the lack of detailed demographic and disability data for excluded participants prevents confirmation of whether lower scan quality disproportionately affected those with higher disability.

Next, the cross-sectional nature of our study prevents an assessment of OCTA’s utility in tracking disease progression over time. Longitudinal studies will be necessary to determine the predictive value of OCTA for disease worsening in MS. Additionally, our healthy control cohort did not undergo MRI brain imaging, limiting comparisons between OCTA-MRI associations in patient versus controls. Investigating these differences in future studies could help delineate OCTA’s specificity in reflecting MS-related neurodegenerative processes.

Our cohort primarily consisted of patients with relapsing-remitting MS on high-efficacy treatment, meaning differences between relapsing and progressive phenotypes could not be analysed, and the study was not designed to assess treatment-class–specific effects. Larger studies focusing on progressive patients would be valuable in clarifying potential disparities in retinal microvasculature associations.

We utilized OCT criteria to define optic neuritis status rather than relying on medical records, as patient recollection was not always consistent. As the aim of this study was to explore the relationship between OCTA and MS biomarkers, fully excluding potential ON effects was not critical in this exploratory context. Nevertheless, potential sources of bias remain, including mild optic neuritis (unilateral or bilateral) that may not meet absolute thresholds. Retinal comorbidities such as glaucoma or vascular disease could also contribute to thinning unrelated to ON, although participants with known retinal pathology were excluded. Finally, although multicollinearity was formally assessed, residual physiological correlations between OCT and OCTA metrics cannot be entirely excluded.

Additionally, cardiovascular risk factors were not commonly present in our cohort, limiting our ability to explore their impact on OCTA metrics. Since cardiovascular comorbidities may contribute to vascular abnormalities, future research should assess their influence on OCTA findings in MS. In addition, not all excluded participants were systematically recorded, which prevented comprehensive demographic or disability comparisons and may introduce the possibility of exclusion bias.

Next, we utilized Ishihara Plates to assess colour vision deficiencies. While this test is convenient for clinical application, it is primarily designed for detecting red-green deficiencies and is less comprehensive than alternative methods such as the Hardy-Rand-Rittler test or Farnsworth-Munsell 100 Hue test. Incorporating these more robust assessments in future studies could provide a more nuanced understanding of colour vision impairments in MS.

Finally, our foundational work suggests possible mechanisms for regional brain atrophy based on associations with OCTA and OCT. The differential distribution of visual cortices introduces a degree of interpretive complexity. Whether the observed regional vulnerability reflects a structural gradient, a network-based phenomenon, the effects of retrograde neurodegeneration or shared metabolic demand in regions within the visual system remains an open question. Future studies incorporating advanced imaging techniques, including functional imaging, brain network and perfusion studies and voxel-based analysis can help clarify this further.

## Conclusion

We conducted a cross-sectional study on a large cohort of predominantly relapsing MS patients and identified unique associations between OCTA-derived retinal microvasculature metrics and regional brain volumes. Our findings suggest distinct pathological mechanisms underlying brain damage and visual loss in MS, as reflected by OCTA and OCT.

In summary, OCTA may serve as a valuable proxy biomarker for brain damage, potentially reflecting broader cerebral hypoperfusion or metabolic supply-demand mismatch in key visual processing centres. In contrast, OCT, particularly pRNFL metrics, may better capture broader neurodegenerative mechanisms, including anterograde or Wallerian degeneration resulting from axonal loss and disrupted connectivity between brain regions. While causality remains to be established, our findings reinforce the added value of OCTA alongside OCT in providing new insights into neurodegenerative processes including neurovascular coupling and regional brain vulnerability, which can form the foundation of future advanced imaging studies and in monitoring MS patients in clinical practice.

## Supplementary Material

fcag007_Supplementary_Data

## Data Availability

Fully anonymized data will be available from the corresponding author upon reasonable request. R code generated for data analysis for this manuscript has been made available via a public GitHub repository accessible through the following link: https://github.com/charmaineyam/OCTA-Volumetrics-MS-code-2025-10-BrainComms.git
