# Supplementary material for: Optical coherence tomography angiography reveals insights into complementary vascular and neurodegenerative mechanisms in multiple sclerosis
Source: Brain Commun. 2026 Jan 10;8(1):fcag007. doi: 10.1093/braincomms/fcag007 (PMC12835921; doi:10.1093/braincomms/fcag007)
Supplement: fcag007_Supplementary_Data [file fcag007_supplementary_data.docx]

**Supplementary Section**

**Supplementary Table 1: Types of DMT switches in non-naïve patients**

| **DMT switch** | **Number of patients (n=139)** |
| --- | --- |
| **DMT escalation**  Dimethyl fumarate-🡪 Ocrelizumab/Ofatumumab  Dimethyl fumarate-🡪 Cladribine  Dimethyl fumarate🡪 fingolimod  Glatiramer acetate -🡪 Ocrelizumab/Ofatumumab  Glatiramer acetate -🡪 Cladribine  Interferon beta 1a-🡪 Ocrelizumab/ofatumumab  Interferon beta 1a 🡪 Cladribine  Teriflunomide🡪 Ofatumumab  **DMT de-escalation**  Ocrelizumab-🡪 Glatiramer acetate  **Non-high efficacy to Non- high efficacy switch**  Dimethyl fumarate🡪 Glatiramer acetate  Glatiramer acetate -🡪 Dimethyl fumarate  Glatiramer acetate -🡪 Interferon beta 1a  Glatiramer acetate -🡪 Teriflunomide  Interferon beta 1a switch formulations  **High efficacy to high-efficacy DMT switch**  Ocrelizumab-🡪Ofatumumab  Ofatumumab-🡪 Ocrelizumab  Alemtuzumab-🡪 Ocrelizumab  Cladribine-🡪 Ocrelizumab/Ofatumumab  Fingolimod-🡪 Ocrelizumab/Ofatumumab  Natalizumab-🡪 Ocrelizumab/Ofatumumab  Ocrelizumab-🡪 Alemtuzumab  Ocrelizumab-🡪 Cladribine  Rituximab-🡪 ofatumumab  Autologous stem cell transplantation-🡪 Ocrelizumab | **48**  **1**  **1**  **17**  **2**  **13**  **3**  **1**  **1**  **4**  **2**  **1**  **1**  **3**  **15**  **1**  **1**  **3**  **8**  **9**  **1**  **1**  **1**  **1** |

**Supplementary Table 2: Summary of OCTA and OCT metrics**

| **OCT/A metric** | **Healthy control (emmean, CI)**  **N= 147 eyes** | **MS- unaffected (emmean, CI)**  **N=505 eyes** | **MS- ON eyes (emmean, CI)**  **N= 98 eyes** | **Estimated marginal means difference (1^st^ group- 2^nd^ group)** | **Confidence Intervals of the estimated differences** | **p-values** |
| --- | --- | --- | --- | --- | --- | --- |
| VAD (%) | 35.40 (34.8-36.0) | 34.10 (33.8-34.4) | 29.50 (29.0-30.0) | NON-HC: -1.28  ON-NON: -4.63  ON-HC: -5.90 | -0.49, – 2.06  -4.02, – 5.24  -4.97, – 6.83 | **0.0004***  **<0.0001* <0.0001*** |
| VLD – pixels (mm^-1^) | 6.06 (5.94-6.17) | 5.79 (5.73-5.86) | 4.93 (4.82-5.03) | NON-HC: -0.27  ON-NON: -0.87  ON-HC: -1.13 | -0.11, – 0.42  -0.74, – 0.99  -0.95, – 1.32 | **0.0002***  **<0.0001***  **<0.0001*** |
| Fractal dimension (1)‡ | 1.619 (1.615-1.623) | 1.611 (1.609-1.613) | 1.580 (1.576-1.584) | NON-HC: -0.0081  ON-NON: -0.031  ON-HC: -0.039 | -0.003, – 0.013  -0.027, – 0.036  -0.033, – 0.046 | **0.0007***  **<0.0001***  **<0.0001*** |
| mGCIPL (µm) | 80.4 (78.9-81.9) | 75.9 (75.1- 76.8) | 63.3 (62.1-64.6) | NON-HC: -4.44  ON-NON: -12.60  ON-HC: -17.04 | - 6.44, -2.44  - 13.99, -11.21  - 19.35, -14.74 | **<0.001***  **<0.0001***  **<0.0001*** |
| pRNFL (µm) | 100.7 (98.6- 102.9) | 95.5 (94.3-96.6) | 80.0(78.3-81.8) | NON-HC: -5.26  ON-NON: -15.42  ON-HC: -20.68 | - 8.16, -2.35  -17.28, -13.55  – 23.97, -17.39 | **0.0001***  **<0.0001***  **<0.0001*** |
| mINL (µm) | 37.4 (36.9-38.0) | 37.8(37.5-38.1) | 38.6 (38.2-38.9) | NON-HC: +0.34  ON-NON: +0.80  ON-HC: +1.14 | -0.38, 1.06  0.48, 1.12  0.37, 1.91 | 0.51  **<0.0001***  **0.0016*** |

Baseline effects (reference categories and intercepts) reported

 †Regression models corrected for age and sex

‡ Unit of 1 indicates dimensionless

MS= multiple sclerosis, HC= healthy control, ON= optic neuritis, NON= non-optic neuritis

mGCIPL= macular ganglion cell inner plexiform layer, pRNFL= peripapillary retinal nerve fibre layer, mINL= macular inner nuclear layer

**Supplementary Table 3: Linear mixed models exploring the relationship between non-visual pathway cortices and OCT/A**

| **OCT/A metric** | **β coefficient** | **Standard error** | **95% CI** | **p- value** |
| --- | --- | --- | --- | --- |
| ***Vessel area density (VAD)***  -Superior temporal gyrus  -Postcentral gyrus (parietal)  -Frontal pole  -Medial frontal cortex | 0.10  0.22  0.44  0.51 | 0.12  0.092  0.28  0.35 | -0.13 to 0.34  0.037 to 0.40  -0.11 to 0.98  -0.18 to 1.19 | 0.39  0.019  0.11  0.15 |
| ***Vessel length density (VLD)***  -Superior temporal gyrus  -Postcentral gyrus (parietal)  -Frontal pole  -Medial frontal cortex | 0.022  0.043  0.095  0.10 | 0.023  0.018  0.054  0.068 | -0.024 to 0.069  0.0074 to 0.078  -0.011 to 0.20  -0.034 to 0.23 | 0.34  0.018  0.078  0.14 |
| ***Fractal dimension (FD)***  -Superior temporal gyrus  -Postcentral gyrus (parietal)  -Frontal pole  -Medial frontal cortex | 0.0005  0.0013  0.0028  0.0032 | 0.00079  0.00061  0.0018  0.0023 | -0.001 to 0.0021  0.000085 to 0.0025  -0.000076 to 0.0064  -0.0013 to 0.0077 | 0.50  0.036  0.12  0.17 |
| ***mGCIPL***  -Superior temporal gyrus  -Postcentral gyrus (parietal)  -Frontal pole  -Medial frontal cortex | 0.49  0.31  0.60  0.43 | 0.31  0.24  0.71  0.89 | -0.11 to 1.09  -0.15 to 0.78  -0.80 to 2.0  -1.33 to 2.19 | 0.11  0.19  0.40  0.63 |
| ***pRNFL***  -Superior temporal gyrus  -Postcentral gyrus (parietal)  -Frontal pole  -Medial frontal cortex | 0.64  0.62  1.71  1.15 | 0.44  0.34  1.01  1.27 | -0.22 to 1.50  -0.041 to 1.29  -0.27 to 3.70  -1.35 to 3.65 | 0.15  0.066  0.090  0.37 |

Baseline effects (reference categories and intercepts) reported

† Parcellated volumes corrected for grey matter volume.

 ‡ All models corrected for ON status (interaction factor), age, sex, disease duration and proportion of disease duration on DMT

*Statistical significance defined as ≤0.01

Interaction plots showing how the slopes of association between Expanded Disability Status Scale (EDSS) and A) Macular ganglion cell inner plexiform layer (mGCIPL) thickness and B) Vessel area density changes with increasing disease duration. Associations were estimated using linear mixed models including an interaction term between EDSS and disease duration. Sample size: n= 323 patients.

Abbreviations: EDSS= Expanded Disability Status Scale; mGCIPL= macular ganglion cell inner plexiform layer.


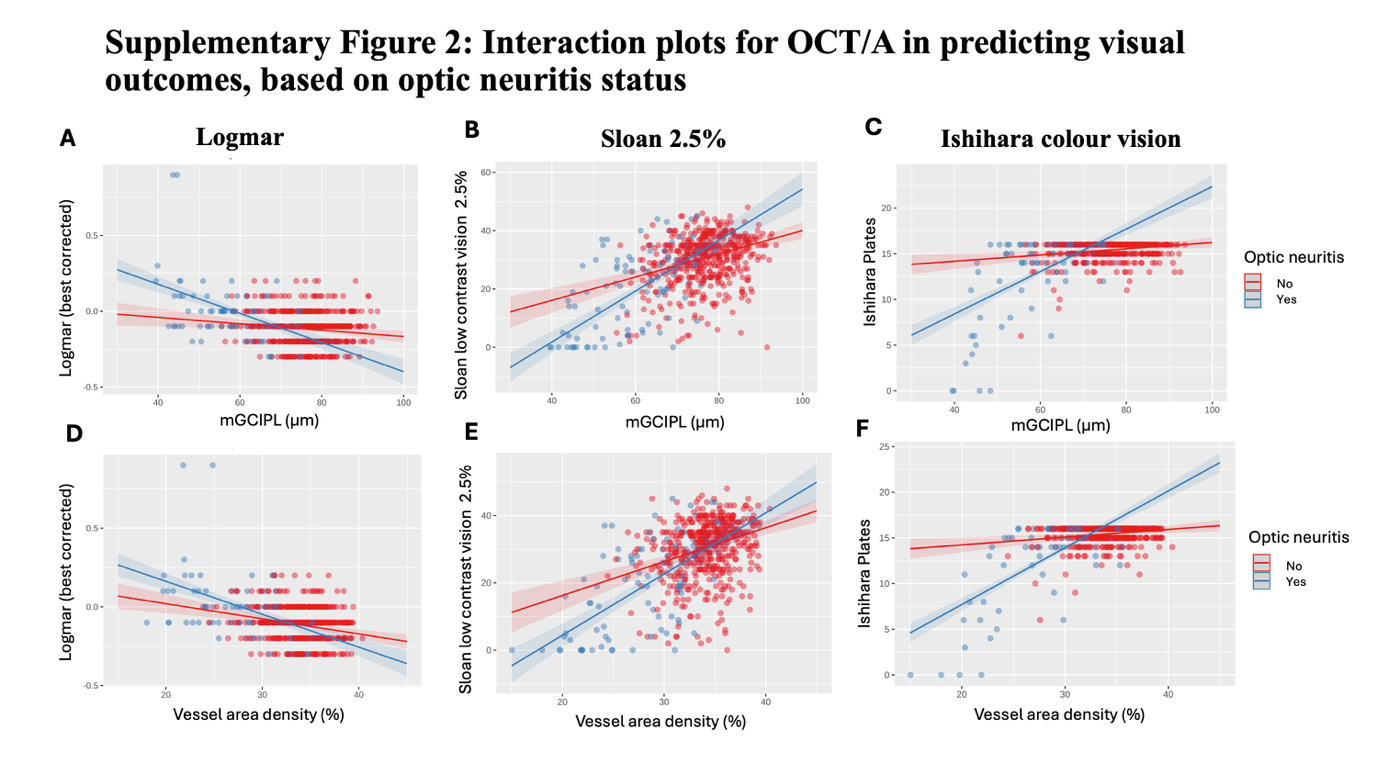


Interaction plots showing how the slopes of association between optical coherence tomography/angiography (OCT/A) in predicting visual outcomes change between optic neuritis (blue line) and non-optic neuritis eyes (red line).

A) Macular ganglion cell inner plexiform layer (mGCIPL) thickness vs Logmar B) mGCIPL thickness vs Sloan 2.5% low contrast visual acuity C) mGCIPL thickness vs Ishihara plates (out of 16)

D) Vessel area density (VAD) vs Logmar E) VAD vs Sloan 2.5% low contrast visual acuity F) VAD vs Ishihara plates (out of 16)

Slopes were estimated using linear mixed models using an interaction term between ON status and each OCT/A metric. The optic neuritis × OCT interaction was statistically significant for all plots shown (p<0.01).

Sample size: n= 321 patients for Logmar and Sloan 2.5% low contrast visual acuity models

n= 296 patients for Ishihara colour vision models

Each datapoint represents a measure taken from an individual eye per patient.

**Supplementary Table 4: Linear mixed models exploring the relationship between Visual scores, EDSS and OCT/A with DMT covariate (proportion of disease duration on DMT vs proportion of disease duration on high-efficacy DMT)**

| **Clinical outcome** | **DMT covariate** | **β coefficient** | **Standard error** | **95% CI** | **p- value** |
| --- | --- | --- | --- | --- | --- |
| ***Logmar***†  VAD  VAD  mGCIPL  mGCIPL  pRNFL  pRNFL | % DMT  % HE-DMT  % DMT  % HE-DMT  % DMT  % HE-DMT | -0.0096  -0.0098  -0.0021  -0.0021  -0.00092  -0.0010 | 0.0021  0.0021  0.00081  0.00080  0.00060  0.00060 | -0.014 to -0.0055  -0.014 to -0.0056  -0.0037 to -0.00051  -0.0037 to -0.00056  -0.0021 to 0.00026  -0.0022 to 0.00018 | **<0.0001***  **<0.0001***  **0.010***  **0.010***  0.12  0.096 |
| ***Sloan 2.5% LCVA***†  VAD  VAD  mGCIPL  mGCIPL  pRNFL  pRNFL | % DMT  % HE-DMT  % DMT  % HE-DMT  % DMT  % HE-DMT | 1.01  1.02  0.40  0.41  0.19  0.20 | 0.15  0.16  0.058  0.058  0.043  0.043 | 0.70 to 1.31  0.72 to 1.33  0.29 to 0.51  0.30 to 0.52  0.11 to 0.28  0.12 to 0.29 | **<0.0001***  **<0.0001***  **<0.0001***  **<0.0001***  **<0.0001***  **<0.0001*** |
| ***Ishihara colour vision***†  VAD  VAD  mGCIPL  mGCIPL  pRNFL  pRNFL | % DMT  % HE-DMT  % DMT  % HE-DMT  % DMT  % HE-DM | 0.084  0.086  0.034  0.035  0.020  0.020 | 0.029  0.029  0.011  0.011  0.0087  0.0086 | 0.028 to 0.14  0.030 to 0.14  0.011 to 0.057  0.012 to 0.057  0.0025 to 0.037  0.0030 to 0.037 | **0.004***  **0.003***  **0.003***  **0.003***  0.03  0.02 |
| ***EDSS***‡  VAD  VAD  mGCIPL  mGCIPL  pRNFL  pRNFL | % DMT  % HE-DMT  % DMT  % HE-DMT  % DMT  % HE-DMT | -0.51  -0.51  -1.52  -1.53  -1.37  -1.40 | 0.15  0.15  0.40  0.40  0.56  0.56 | -0.81 to -0.21  -0.81 to -0.21  -2.30 to -0.75  -2.31 to -0.75  -2.48 to -0.27  -2.51 to -0.29 | **0.001***  **0.001***  **0.0001***  **0.0001***  0.015  0.014 |

†Models corrected for age, gender, disease duration (DD) and proportion of disease on DMT and optic neuritis status (as an interaction term).

‡ Models corrected for age, sex, proportion of disease on DMT, optic neuritis status and disease duration (DD) (as an interaction term).

*Statistical significance defined as ≤0.01 and shown in **bold**

High-efficacy DMT (HE-DMT)

**Supplementary Table 5: Linear mixed models exploring the relationship between MRI metrics and OCTA**

| **OCT/A metric** | **DMT covariate** | **β coefficient** | **Standard error** | **95% CI** | **p- value** |
| --- | --- | --- | --- | --- | --- |
| ***Vessel area density (VAD)***  -T2 lesion volume  -T2 lesion volume  -White matter volume  -White matter volume  -Deep grey matter volume  -Deep grey matter volume  -Cortical grey matter volume  -Cortical grey matter volume  -Lesion number  -Lesion number | % DMT  % HE-DMT  % DMT  % HE-DMT  % DMT  % HE-DMT  % DMT  % HE-DMT  % DMT  % HE-DMT | -0.050  -0.050  0.020  0.020  0.050  0.060  0.040  0.040  -0.015  -0.015 | 0.022  0.022  0.011  0.011  0.089  0.089  0.014  0.014  0.0062  0.0061 | -0.094 to -0.0068  -0.093 to -0.0066  -0.0057 to 0.037  -0.0052 to 0.037  -0.12 to 0.23  -0.12 to 0.23  0.012 to 0.067  0.013 to 0.069  -0.027 to -0.0024  -0.027 to -0.0027 | 0.023  0.024  0.15  0.15  0.54  0.54  **0.0055***  **0.0046***  0.019  0.017 |
| ***(i) Anterior pathway***  -Optic chiasm  -Optic chiasm  -Thalamus  -Thalamus  ***(ii) Primary visual cortex***  -Occipital pole  -Occipital pole  -Calcarine cortex  -Calcarine cortex  ***(iii) Visual association cortices***  ***Occipital lobe***  -Superior occipital gyrus  -Superior occipital gyrus  -Inferior occipital gyrus  -Inferior occipital gyrus  -Cuneus  -Cuneus  -Occipital fusiform gyrus  -Occipital fusiform gyrus  -Lingual gyrus  -Lingual gyrus  ***Non-occipital lobe***  -Inferior temporal gyrus  -Inferior temporal gyrus  -Superior parietal gyrus  -Superior parietal gyrus | % DMT  % HE-DMT  % DMT  % HE-DMT  % DMT  % HE-DMT  % DMT  % HE-DMT  % DMT  % HE-DMT  % DMT  % HE-DMT  % DMT  % HE-DMT  % DMT  % HE-DMT  % DMT  % HE-DMT  % DMT  % HE-DMT  % DMT  % HE-DMT | 25.74  26.04  0.52  0.52  0.66  0.65  0.50  0.49  0.44  0.43  0.28  0.28  0.36  0.35  0.51  0.50  0.25  0.24  0.22  0.21  0.26  0.26 | 17.94  17.94  0.14  0.15  0.23  0.23  0.14  0.14  0.19  0.19  0.11  0.11  0.13  0.13  0.18  0.18  0.098  0.098  0.087  0.087  0.10  0.10 | -9.60 to 61.07  -9.28 to 61.36  0.24 to 0.81  0.23 to 0.80  0.21 to 1.11  0.20 to 1.10  0.21 to 0.78  0.21 to 0.78  0.065 to 0.81  0.057 to 0.81  0.066 to 0.49  0.062 to 0.49  0.096 to 0.62  0.092 to 0.62  0.17 to 0.86  0.15 to 0.85  0.054 to 0.44  0.049 to 0.44  0.046 to 0.39  0.042 to 0.38  0.059 to 0.46  0.053 to 0.46 | 0.15  0.15  **0.0004***  **0.0004***  **0.0040***  **0.0046***  **0.0007***  **0.0008***  0.022  0.024  **0.010***  0.012  **0.0077***  **0.0084***  **0.0039***  **0.0048***  0.013  0.014  0.013  0.015  0.012  0.014 |

 † Main white and deep grey matter volumes corrected for TIV.

 ‡ metrics based on brain volumes/T2 lesion volume in millilitres

 # Parcellated volumes corrected for grey matter volume, except for optic chiasm.

 ## All models corrected for ON status (interaction factor), age, sex, disease duration and proportion of disease duration on DMT

*Statistical significance defined as ≤0.01 and shown in **bold**

**Supplementary Table 6: MRI AICc comparison of disease duration vs optic neuritis interactions for VAD and GCIPL**

| **MRI metric** | **AICc for Model with MRI × optic neuritis interaction** | **AICc Model with MRI × disease duration interaction** | **ΔAIC (ON – Disease Duration)** |
| --- | --- | --- | --- |
| **VAD**  -Cortical grey matter  -White matter  -Thalamus  -Occipital pole  -Occipital fusiform gyrus  -Lingual gyrus | 2424.98  2433.41  2423.49  2426.28  2421.30  2424.20 | 2429.79  2438.10  2427.82  2431.48  2435.37  2438.79 | -4.81  -4.69  -4.33  -5.2  -14.07  -14.59 |
| **GCIPL**  -Cortical grey matter  -White matter  -Thalamus  -Occipital pole  -Occipital fusiform gyrus  -Lingual gyrus | 3248.28  3257.31  3247.43  3252.94  3253.26  3255.83 | 3253.19  3262.84  3250.38  3259.48  3263.39  3264.17 | -4.91  -5.53  -2.95  -6.54  -10.13  -8.34 |

**Supplementary Analysis 1: Additional analysis to explore potential mechanisms of colour vision deficits and the contributions of central mechanisms**

In patients with bilateral non-ON eyes (n = 185 pairs), Ishihara colour vision scores were highly similar between left and right eyes, with most eyes achieving the maximum score of 16 plates, resulting in a skewed distribution. A paired Wilcoxon signed-rank test confirmed no significant difference between eyes (V = 2663, 95% CI −0.15 to 0.18, p = 0.90), supporting the notion that colour vision deficits in these eyes are largely symmetrical and consistent with potential central contributions. Only participants with both left and right eye measurements were included in the paired Wilcoxon signed-rank test (n = 185 pairs). The graph shows all available eyes (n = 191), but incomplete pairs were excluded from the analysis.

Next, we tested interactions between OCT-A and OCT metrics and MRI volumetrics in colour vision–processing brain areas (lingual gyrus, occipital fusiform gyrus, and inferior temporal gyrus), comparing nested models with and without the interaction term using ANOVAs. For GCIPL and pRNFL, the interaction term was nominally significant for the inferior temporal gyrus model (p < 0.05), but model comparisons indicated that the simpler non-interaction model provided a better fit for both metrics. In all other models, including those with OCT-A and those examining OCT vs lingual or occipital fusiform gyrus, no significant interactions were observed, and the non-interaction models also consistently had lower AIC. Together, these results suggest that retinal and cortical contributions to colour vision deficits act independently rather than synergistically.

Supplementary Analysis 1 is included for completeness but is not referenced in the main text.

**Supplementary analysis 2: ROC analyses: Investigating the ability of OCT/OCTA metrics in discriminating non-optic neuritis eyes from healthy control eyes**

Receiver operating characteristic (ROC) analyses were performed to explore the ability of OCT and OCTA parameters to discriminate non-ON eyes from healthy control eyes. To account for inter-eye correlation, we averaged bilateral eyes per patient for both non-ON eyes (n=202 patients) and healthy controls (n= 67) and adjusted the models for age and sex to avoid potential confounding discrimination metrics. AUC values were modest across modalities, ranging from 0.626 (pRNFL) to 0.673 (GCIPL). OCTA metrics (VAD, VLD, FD) showed consistent but moderate separation across the ROC space (AUC VAD: 0.641, VLD: 0.646, FD: 0.646), while OCT curves (GCIPL, pRNFL) deviated from the diagonal primarily at higher specificity thresholds, with limited discrimination at high sensitivity. These patterns align with our mechanistic interpretation: OCTA captures diffuse vascular alterations that provide modest but uniform discrimination, whereas OCT reflects later neuroaxonal loss most evident at higher specificity.

**Supplementary Figure 3: ROC Curves of OCT and OCTA metrics for discriminating non-ON eyes from healthy control**

| **Metric** | **AUC (95% CI)** |
| --- | --- |
| A) VAD  B) VLD  C) FD  D) mGCIPL  E) pRNFL | 0.641 (0.567-0.714)  0.646 (0.573-0.719)  0.646 (0.572-0.720)  0.673 (0.606-0.739)  0.626 (0.552-0.700) |

ROC curves of Optical coherence tomography and angiography metrics including:

Vessel area density (VAD), Vessel length density (VLD), Fractal dimension (FD), macular ganglion cell inner plexiform layer (mGCIPL) and peripapillary retinal nerve fibre layer (pRNFL) with respective Area under the curves (AUC) shown with 95% confidence intervals (CI).

Sample sizes: Healthy controls (n= 67), Multiple sclerosis patients with bilateral non-optic eyes (n=202).
